# Overexpression of *Tisochrysis lutea* Akd1 identifies a key cold-induced alkenone desaturase enzyme

**DOI:** 10.1038/s41598-018-29482-8

**Published:** 2018-07-25

**Authors:** Hirotoshi Endo, Yutaka Hanawa, Hiroya Araie, Iwane Suzuki, Yoshihiro Shiraiwa

**Affiliations:** 1 Faculty of Life and Environmental Sciences, University of Tsukuba, 1-1-1 Tennodai, Tsukuba, Ibaraki 305-8572 Japan; 2 Present Address: College of Science and Engineering, Kanto Gakuin University, 1-50-1 Mutsuura-higashi, Kanazawa-ku, Yokohama, Kanagawa 236-8501 Japan

## Abstract

Alkenones are unusual long-chain neutral lipids that were first identified in oceanic sediments. Currently they are regarded as reliable palaeothermometers, since their unsaturation status changes depending on temperature. These molecules are synthesised by specific haptophyte algae and are stored in the lipid body as the main energy storage molecules. However, the molecular mechanisms that regulate the alkenone biosynthetic pathway, especially the low temperature-dependent desaturation reaction, have not been elucidated. Here, using an alkenone-producing haptophyte alga, *Tisochrysis lutea*, we show that the alkenone desaturation reaction is catalysed by a newly identified desaturase. We first isolated two candidate desaturase genes and found that one of these genes was drastically upregulated in response to cold stress. Gas chromatographic analysis revealed that the overexpression of this gene, named as *Akd1* finally, increased the conversion of di-unsaturated C_37_-alkenone to tri-unsaturated molecule by alkenone desaturation, even at a high temperature when endogenous desaturation is efficiently suppressed. We anticipate that the *Akd1* gene will be of great help for elucidating more detailed mechanisms of temperature response of alkenone desaturation, and identification of active species contributing alkenone production in metagenomic and/or metatranscriptomic studies in the field of oceanic biogeochemistry.

## Introduction

Alkenones are long-chain unsaturated methyl- or ethyl- ketones, produced by selected haptophyte algae^1–5^. In these species, C_37_-methyl-alkenones, containing two to four *trans*-type carbon double bonds, are the most prominent molecules found. Among them, two- and three-double bond molecules (designated as C_37:2_ and C_37:3_, respectively) have frequently been used for the reconstruction of palaeotemperature, as described below. As the cellular production of alkenones with three double bonds is promoted at low temperature, and *vice versa*, the proportional relation between growth temperature and the unsaturation ratio of C_37_-alkenones: *U*^*k*’^_37_ = C_37:2_/(C_37:2_ + C_37:3_) has been established as a molecular thermometer^6–10^. Such temperature-dependent changes in the unsaturation ratio of alkenones and their abundance in ocean sediments enable us to estimate past sea surface temperatures.

Despite its importance in palaeoclimatology, there is only a limited understanding of alkenone biosynthesis and the metabolism of various derivatives, such as alkenoates, although a hypothesis has been proposed^11,12^. Recently, by performing ^13^C-pulse-chase experiments, we discovered that C_37:3_ is synthesised from C_37:2_ in the alkenone desaturation pathway in response to cold stress^13^. This finding suggests that there is a cold-responsive desaturase enzyme that is the key for the desaturation of C_37_-alkenones.

Desaturation in lipid fatty acyl chains is catalyzed by fatty acid desaturases (FADs) found in almost all organisms^14–16^. Those enzymes are classified into two large phylogenetically independent families; i.e., soluble FADs and membrane-bound FADs. The soluble FADs, known to be located only in the plastid, form a Δ9-carbon unsaturation bond in stearoyl-ACP. On the other hand, the membrane-bound FADs function to introduce an unsaturation bond into acyl lipids or acyl chains of acyl-CoA in either the endoplasmic reticulum (ER) or the plastid. These FADs possess four or more trans-membrane domains and three conserved histidine-rich motifs (His boxes) in a molecule. Additionally, FADs in this family are characterized by the presence of intriguing sequence diversity in comparison with soluble FADs showing high sequence similarities. From this point of view, putative alkenone desaturase(s) could possibly be some variant(s) classified in this family.

In haptophyte algae, several studies have been conducted to ascertain the function of FADs and lipid metabolism in molecular, cellular and genome analyses^17–22^. However, no information is available on desaturases functioning as alkenone desaturases yet although the desaturation reaction of C_37:2_- to C_37:3_-alkenones is already proved by metabolite analysis and ^13^C-tracer experiment^13^. First, this study aimed to identify the gene and enzyme of alkenone desaturase to prove the molecular mechanism involved in desaturation reaction of alkenones. Further, the information will be of importance for better understanding of the metabolic processes for the temperature-dependent change in the alkenone unsaturation index which has been frequently used for the reconstruction of sea surface temperature based on alkenone-producing haptophyte physiology.

In the present study, we identified candidate genes by comparative sequence analysis, and confirmed the function of the gene by the newly established overexpression system. Here we report the first identification and experimental functional analysis of a novel *trans*-desaturase which catalyzes desaturation reaction of alkenone.

## Results

### *Tisochrysis lutea* as a target for alkenone studies

The most extensively studied haptophyte species *Emiliania huxleyi* was recently started to be used for genome analysis and molecular biological studies^21,22^. The species also has frequently been used for studies on the regulation of alkenone production and metabolism by various environmental factors^4,6,13,23,24^. However, *E*. *huxleyi* is now recognized as a species which is difficult to use gene technology because of no successful example of genetic transformation system. We assumed that the difficulty in *E*. *huxleyi* cells may be due to several reasons such as low growth rate, small cell size, and the presence of coccoliths and organic scales covering and guarding cell surface against the treatments of transformation.

**Figure 1:**
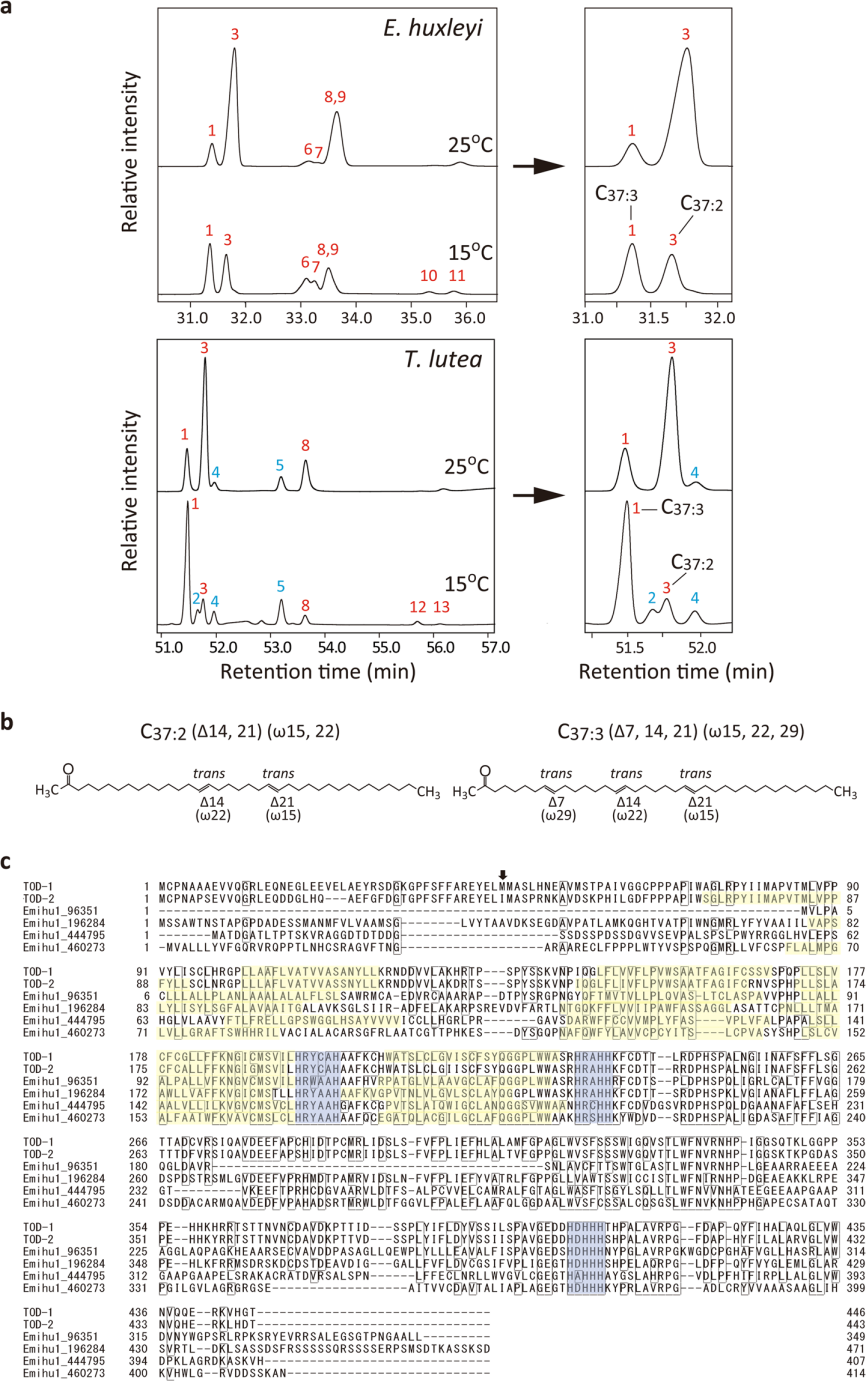
Cold-induced changes in the alkenone profiles of *Emiliania huxleyi* and *Tisochrysis lutea*, and alignment of alkenone desaturase candidates. (**a**) Partial GC-FID chromatograms of alkenones and alkenoates in the wild type cells of *E. huxleyi* and *T. lutea* grown at 25 °C and 15 °C. The figures at the right of arrows are those expanded partially. Peaks: 1, C_37:3_; 2, C_36:3_FAME; 3, C_37:2_; 4, C_36:2_FAME; 5, C_36:2_FAEE; 6, C_38:3_Et; 7, C_38:3_; 8, C_38:2_Et; 9, C_38:2_; 10, C_39:3_Et, 11, C_39:2_Et; 12, C_39:3_; 13, C_39:2_. Red and blue letters represent alkenones and alkenoates, respectively. (**b**) Molecular structures of di- and tri-unsaturated C_37_-alkenones. (**c**) Alignment of the amino acid sequences of alkenone desaturase candidates. TOD-1 and -2: sequences identified in the *T. lutea* transcriptome database^19^. Emihu1_96351, 196284, 444797 and 460273: JGI gene IDs from *E. huxleyi*. The box indicates amino acid residues conserved in more than four sequences. Blue and yellow: conserved His-boxes and putative trans-membrane regions predicted by the SOSUI engine ver. 1.11 (http://harrier.nagahama-i-bio.ac.jp/sosui/), respectively. Arrow: the initiation of the ORF in the vectors pLF17Akd1-F and -R (Fig. 3a).

Among alkenone-producing haptophytes, we selected an alternative alkenone-producing species, *T*. *lutea* T-iso (formerly classified in the genus *Isochrysis* as *I. galbana* T-iso)^25^ for use in this study. The two most abundant di- and tri-unsaturated C_37_-methyl-*n*-ketones (alkenones) were produced by *T*. *lutea* both at 25 °C, near the optimum temperature for growth, and at 15 °C, although the C_37:3_/C_37:2_ ratios differed from those in *E*. *huxleyi* (Fig. 1a,b). These data verify that *T*. *lutea* is a suitable candidate for studying alkenone metabolism.

### Search of alkenone desaturase candidates

Firstly, we isolated 17 FAD-sequences from the genomic database of *E*. *huxleyi*, and the phylogenetic tree was constructed with amino acid sequences of already characterized or annotated desaturases from the pennate diatom *Phaeodactylum tricornutum*^26^ and higher plant *Arabidopsis thaliana* (Supplementary Fig. 1). Among the six clusters, one cluster involved four “orphan” desaturase sequences (i.e., sequences that show high homology to other desaturases already characterised, but are neither annotated nor substrate defined).

By expecting that any of these four desaturases may catalyse the alkenone desaturation reaction, a homology search of the *T*. *lutea* transcriptome database^19,27^ was performed using these four orphan *E*. *huxleyi* sequences. As a result, two homologous protein sequences were identified and tentatively named as *T*. *lutea* orphan desaturase (TOD) -1 and -2, (Fig. 1c). All of these alkenone desaturase candidates share three well-conserved His boxes. Characterizing these genes, the third His box contains three unusual consecutive His residues that are not observed in other typical FADs^14^. In addition, TOD-1 and -2 contain multiple trans-membrane regions, similar to those of membrane-bound FADs^14,15^.

### Cold-induced expression of *TOD-1* and *-2*

In order to examine whether these two genes (*TOD-1* and -*2*) were associated with alkenone desaturation, the effects of temperature on the gene expression as well as cell growth, C_37_-alkenone levels and the C_37:3_/C_37:2_ ratio were determined. *T*. *lutea* cells were grown autotrophically under continuous light at 15, 20, 25, or 30 °C for nine days. The growth rates were similar at 20–30 °C, but low at 15 °C where carbon allocation to the alkenone pool (alkenone/total organic carbon) was also low (Supplementary Fig. 2a).

The contents of individual C_37_-alkenones were separately quantified by gas chromatography-flame ionization detector (GC-FID). In the analysis, the saponification is known to be useful usually to get better quantification of C_37:2_ by removing the overlapped alkenoate C_36:2_ FAME peak. However, the quantification of C_37:3_ and C_37:2_ contents was performed without saponification of the samples in this study, since significant differences in the quantification were not observed, irrespective of saponification, in our separate experiments (Supplementary Fig. 2b).

**Figure 2:**
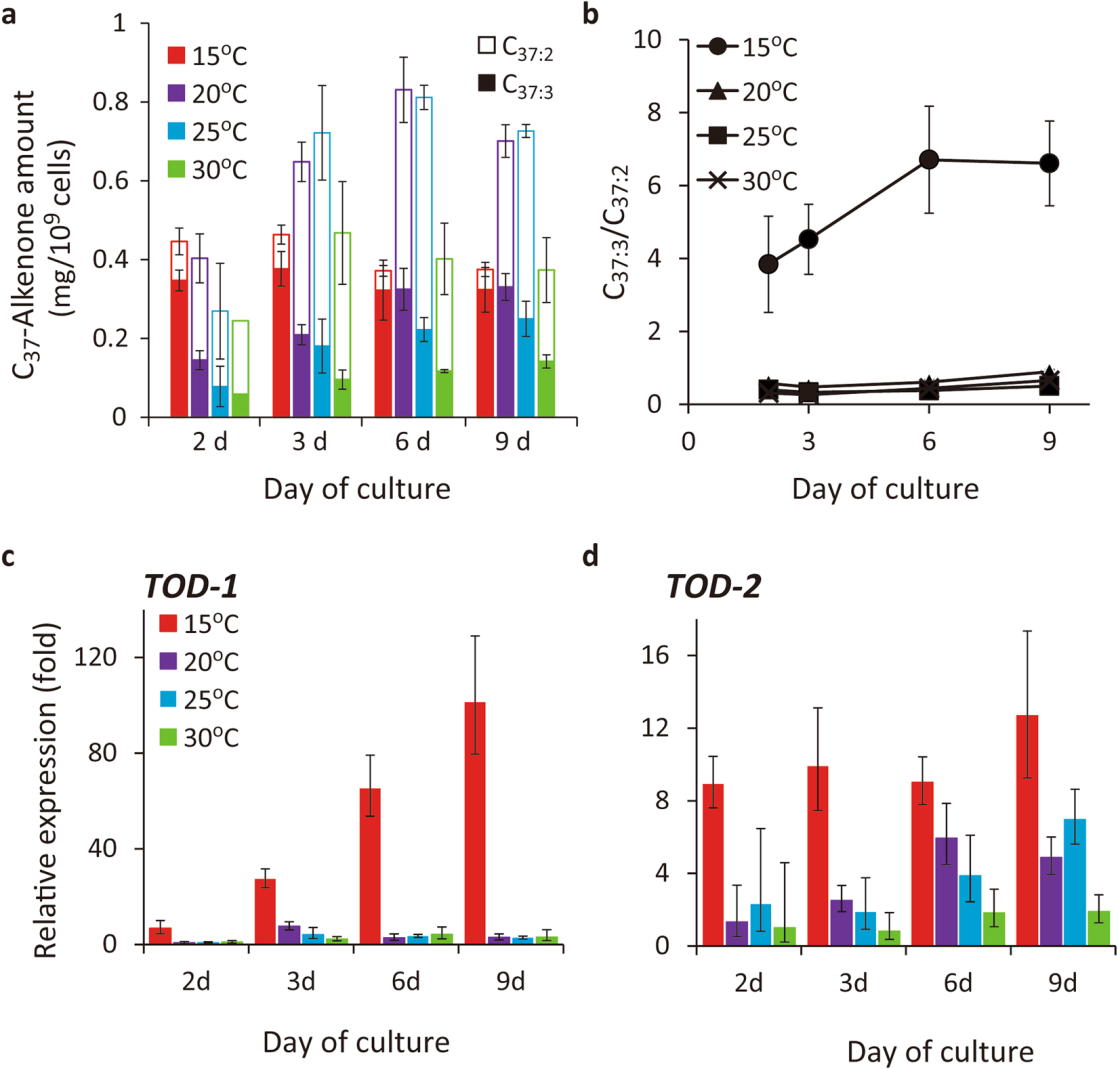
Temperature-dependent changes in alkenone production, unsaturation status, and expression of alkenone desaturase candidate genes during culture in the wild type of *Tisochrysis lutea*. (**a**) Total C_37_-alkenone contents. (**b**) C_37:3_/C_37:2_ ratio. Data: mean ± s.d. (*n* = 4) except on day 2 at 30 °C (*n* = 2). (**c**,**d**) Relative expression of *TOD-1* (**c**) and *-2* (**d**), respectively. The values are normalised to that at 30 °C on day 2 for each gene.

Total C_37_-alkenone contents increased largely at 20 °C and 25 °C during 6 days, increased slightly at 30 °C, but remained constant at 15 °C (Fig. 2a). On the other hand, the C_37:3_/C_37:2_ ratio increased dramatically (3.9–6.5 fold) at 15 °C during first 6 days, but was remained constant at a low value (0.2–0.4 fold) at 20–30 °C (Fig. 2b, Supplementary Fig. 2c). These data indicate that alkenone desaturation is stimulated at low temperatures.

The expression patterns of *TOD-1* and -*2* were clearly different in quantitative real-time PCR analysis. At 15 °C, changes in the expression of *TOD-1* and the C_37:3_/C_37:2_ ratio showed very similar patterns (Fig. 2b,c), whereas the expression level of *TOD-2* was almost constant during the culture (Fig. 2d). In addition, the expression levels of *TOD-1* increased approximately 100-fold from day 2 to day 9, whereas the expression of *TOD-2* increased only 1.1-fold over the same time frame. These data strongly indicate that TOD-1 promoted the desaturation of C_37:2_ to C_37:3_ in response to cold stress and the evidence suggest that TOD-1 is most likely to be an enzyme “alkenone desaturase”. Accordingly, we focused on TOD-1 for the subsequent experiments.

### Establishment of genetic transformation technique for *T. lutea* and its application to functional analysis of *TOD-1*

In haptophyte algae, several FADs have been functionally characterised by using either cyanobacterial or yeast expression systems^17,18,20,22,28^. However, heterologous expression cannot be used for the functional analysis of genes associated with alkenone biosynthesis since alkenones are produced by only five species of haptophytes, but no any other organisms. We, therefore, needed to develop a method to take an alternative approach, such as transformation, to study on the mechanism of alkenone biosynthesis.

Recently, a reliable transformation system has been successfully established for the non-alkenone-producing haptophyte species, *Pleurochrysis carterae*^29^. By introducing the method into the present study, we tried to modify the method and apply it to an alkenone-producing haptophyte *T. lutea*. Finally, we succeeded in establishing the method which is applicable to *T. lutea* and obtained the mutant strains that overexpress *TOD-1* stably (see Methods, Supplementary Fig. 3).

**Figure 3:**
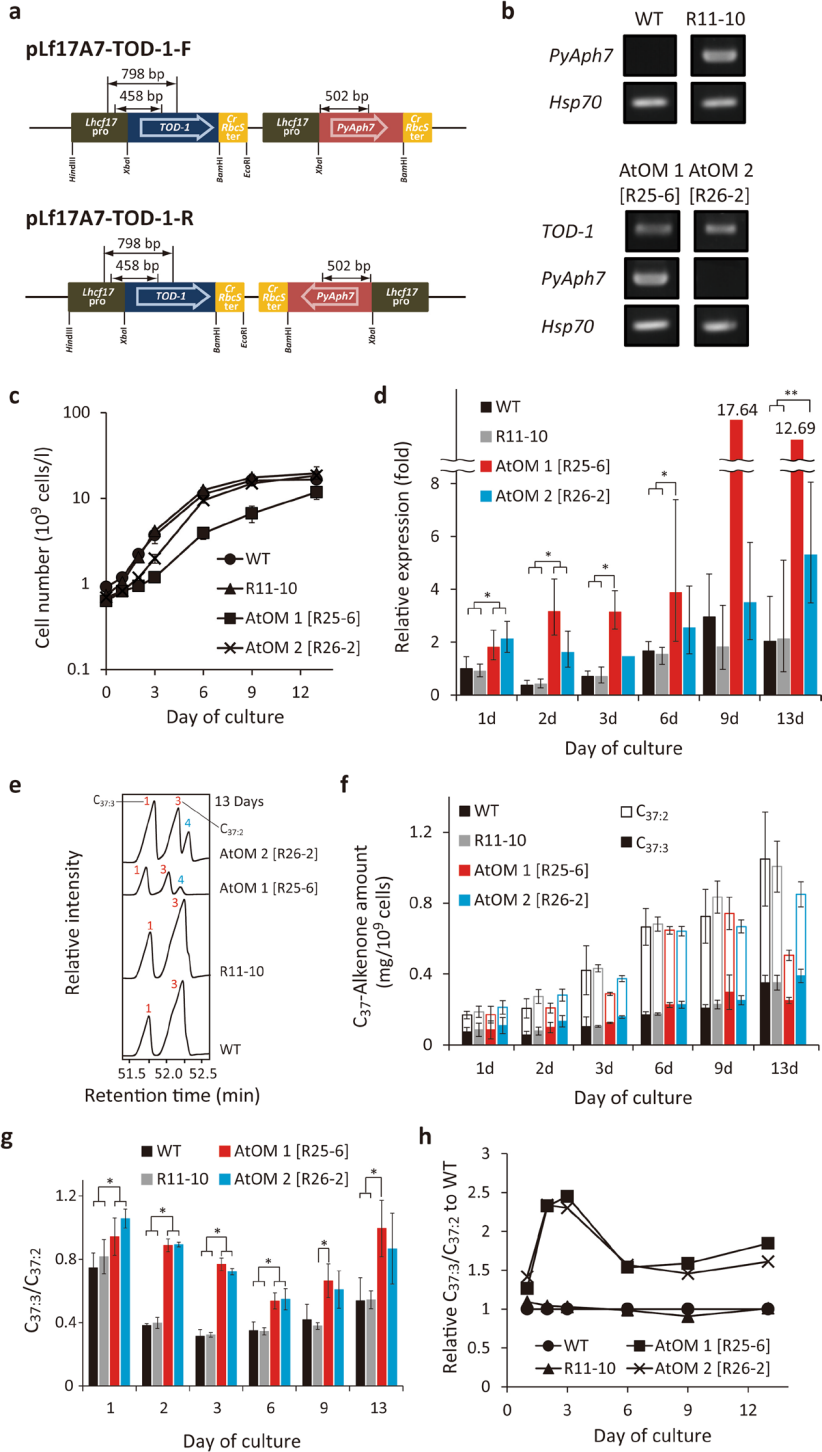
Characterization of the *TOD-1* overexpressing mutants of *Tisochrysis lutea*. (**a**) The expression constructs containing *TOD-1* and *PyAph7* (a codon-modified hygromycin B-resistance gene optimised for GC-rich algae^29^). Double-headed arrows: regions amplified in the genotyping PCR reactions. *Lhcf17*pro: endogenous promoter for *Light-harvesting complex f17* (*Lhcf17*) gene of *T. lutea*. *CrRbcS*ter: terminator from the *ribulose-1*,*5-bisphosphate carboxylase/oxygenase* (*RuBisCO*) small subunit gene from *Chlamydomonas reinhardtii*. (**b**) Genotyping by genomic PCR. WT: wild type. R11-10: a *PyAph7*-harbouring control strain. AtOM 1 [R25-6] and 2 [R26-2]: alkenone desaturase candidate overexpression mutants transformed with vectors pLf17A7-TOD-1-R and -F, respectively. (**c**) Growth profiles of the different strains. (**d**) Expression analysis of *TOD-1*. Relative expression was normalised to the value in WT strain on day 1. Data: mean ± s.d. (*n* = 3) except for AtOM 1 on days 9 (*n* = 2) and 13 (*n* = 1) and AtOM 2 on day 3 (*n* = 2). Significant differences determined by statistical analysis (ANOVA-Tukey-Kramer) are shown (*p < 0.01 and **p < 0.025). (**e**) Partial GC-FID chromatograms on day 13. (**f**) Total C_37_-alkenone contents. Peak numbers and colours are same as shown in Fig. 1a. (**g**) C_37:3_/C_37:2_ ratios. Data: mean ± s.d. (*n* = 3). (**h**) Relative C_37:3_/C_37:2_ ratios normalised to that in WT. Original unmodified images of gel electrophoresis are shown in Supplementary Fig. 7.

To generate these mutant strains, tandem expression vectors containing *TOD-1* and *PyAph7* (a codon-optimised hygromycin B-resistance gene) were constructed (Fig. 3a). The vectors were separately introduced into *T. lutea* cells and successful gene integration was observed in 28 strains (Supplementary Fig. 4). Among these strains, those referred to as R25-6 and R26-2 showed the highest expression of *TOD-1* and had potent activity of C_37:3_ biosynthesis converted from C_37:2_ by desaturation reaction (the procedure used to screen the mutants is summarised in Supplementary Fig. 5). From such evidences, these two strains were named Alkenone *trans*-desaturase Overexpressing Mutant (AtOM) 1 and 2, and were selected to be used for further analyses.

The two *TOD-1* overexpressing strains (AtOM 1 and 2), as well as two control strains (wild type (WT) and R11-10 expressing *PyAph7* but not *TOD-1*), were cultured at 30 °C (Fig. 3b). At 30 °C, the expression of endogenous *TOD-1* is already proved to be down-regulated, and accordingly the C_37:3_/C_37:2_ ratio is maintained at a low level (see Fig. 2). Although the growth of AtOM 1 was slightly slower than that of the other strains (Fig. 3c), enhanced expression of *TOD-1* in both, AtOM 1 and 2, strains was observed over the culture period (Fig. 3d). An increase in the unsaturation ratio of the C_37_-alkenones (i.e., shown by a high C_37:3_/C_37:2_ ratio), due to both an increase in C_37:3_ production and a decrease in C_37:2_ production, was clearly observed in both *TOD-1* overexpressing strains compared with that in the WT and R11-10 strains (Fig. 3e, Supplementary Fig. 6a).

The total C_37_-alkenone content of each of the four strains increased moderately in the cultures over time, with the exception of AtOM 1, in which the alkenone content reached the maximum on day 9 (Fig. 3f, Supplementary Fig. 6b). The C_37:3_/C_37:2_ ratio, used as a parameter of unsaturation degree, was higher across the complete time course in both AtOM strains compared with that in the two control strains, even at 30 °C (Fig. 3g). The quantification of C_37:2_ may contain some errors by the contamination of alkenoates into C_37:2_ peak in GC-FID chromatograms. However, the difference in the C_37:3_/C_37:2_ ratio was estimated as 5.6~14.5% in both WT and the AtOM mutants according to examination of GC-FID analysis of alkenones before and after saponification (Supplementary Fig. 2b). When the C_37:3_/C_37:2_ ratio was normalised to that of the WT at each time point, the ratio in the AtOM strains was approximately 1.5 to 2.5-fold higher (Fig. 3h). These data indicate that the overexpression of the *TOD-1* gene promotes the desaturation of C_37:2_ to produce C_37:3_. On the basis of this evidence, we designated this gene as *Alkenone desaturase 1* (*Akd1*).

### Primary structural features of Akd1

**Figure 4:**
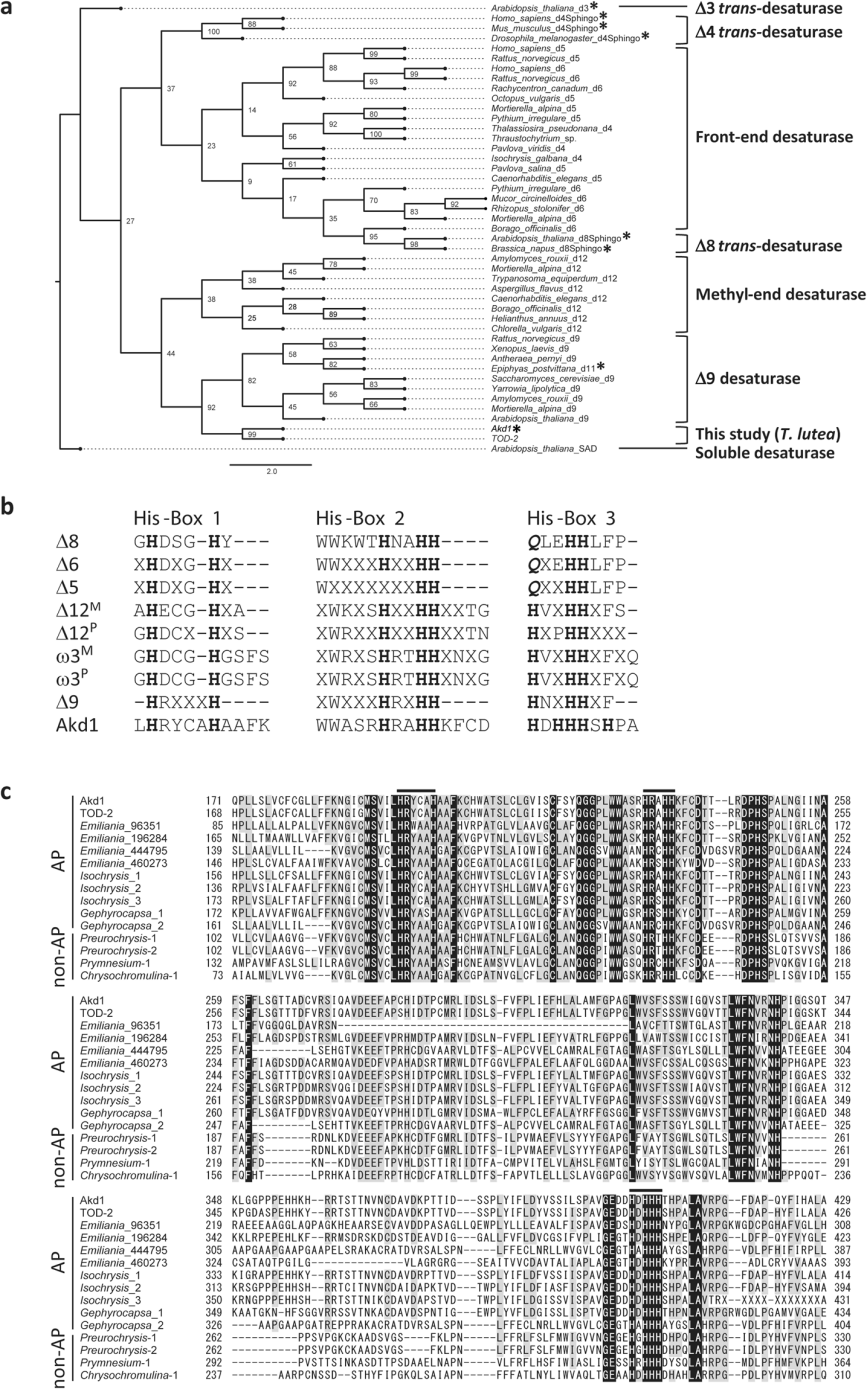
The maximum likelihood phylogenetic tree and consensus sequences of His boxes in various desaturases. (**a**) The phylogenetic position of Akd1 annotated as alkenone desaturase among various types of desaturase. *trans-desaturases identified by functional characterization. The scientific name is followed by a simplified regioselectivity of each enzyme (i.e., ∆x is designated as dx). (**b**) Alignment of consensus sequences of the His boxes in Akd1 and other fatty acid desaturases (revised from Alonso *et al*.^16^). X: Arbitrary amino acid residue. Bold and *italic*: Conserved amino acid residues of histidine and glutamine, respectively. M and P: microsome- and plastid-located enzyme, respectively. (**c**) Alignment of partial sequences of Akd1-homologues. Black and grey: amino acid residues conserved in all and more than six sequences, respectively. Bar: His box. AP and non-AP represent alkenone producer and non-alkenone producing haptophytes, respectively.

A phylogenetic analysis performed on various desaturases from a wide range of taxa suggests that Akd1 is closely related to the ∆9 desaturase family, which is assumed to be the ancestor of all the membrane-bound FAD families (Fig. 4a)^15,16^. The insertion of four amino acid residues between two conserved His residues in the first His box is a remarkable characteristics in both Akd1 and other known ∆9 FADs (Fig. 4b). We also found that *Akd1*-homologous genes are conserved in other haptophyte algae. As shown in Fig. 4c, these sequences show high similarity, especially around the His boxes.

## Discussion

In the present study, we present experimental evidences showing that Akd1 catalyses the desaturation reaction of responsible for converting C_37:2_ to C_37:3_ by analysing alkenones in the overexpression mutant strains, AtOMs.

The function of alkenones as a storage neutral lipid is similar to other neutral lipids such as triacylglycerol (TAG) produced by many other microalgae, but the biosynthetic pathway and physiological response of alkenones to changes in environmental factors are different from TAG. Actually, in *E*. *huxleyi* which is not a TAG producer, alkenones are used as the major carbon and energy storage compounds since neutral polysaccharides are not produced much^24^. Alkenones are stored in a specific organelle named as the alkenone body, which is assumed to be extruded from the endoplasmic reticulum (ER) in the proteomic analysis of the alkenone body isolated from *T*. *lutea*^19^. Although the alkenone body is expected as one of candidate for localization, Akd1 was not found in the list of major proteins in our previous proteomics analysis^19^. This may be due to that the cells used were grown at 20 °C and *Akd1* is inducible at 15 °C (see Fig. 2). The molecule of Akd1 has no translocation signal sequences such as chloroplast-localization or ER-retrieval signals. Further analysis is needed for determining the localization of Akd1. Additionally, further necessary studies to concrete our present evidence are the analysis of Akd1 protein level by Western blot analysis, and ^13^C-tracer-experiment to confirm the enzymatic conversion of C_37:2_ to C_37:3_ in kinetic analysis by the method used in our previous study^13^.

The positions of the *trans*-carbon double bonds in alkenone molecules are rigidly fixed according to previous literatures^1,2,6,7^. The *trans*-double bonds are located at the positions, ∆14 and ∆21, and the third double bond is added at the ∆7 position in C_37:3_ by desaturation reaction (see Fig. 1b). Although the present study did not clarify the precise position of the third double bond, Akd1 is likely to be the ∆7 *trans*-desaturase.

The phylogenetic origin of *trans*-desaturases is complex since most double bonds in fatty acyl chains are in the *cis* configuration. The ∆3 and ∆11 *trans*-desaturases have been identified in *Arabidopsis thaliana*^30^ and the moth, *Epiphyas postvittana*^31^, respectively. However, both of these FADs are phylogenetically distant from each other (Fig. 4a). Some members of the sphingolipid desaturase family, namely ∆4 desaturases in animals^32^ and ∆8 desaturases in land plants^33^ are also *trans*-desaturases. However, Akd1 is different from none of already known desaturases in the phylogenetic tree (Fig. 4), indicating that Akd1 is a novel *trans*-desaturase.

We also found that Akd1-like protein is also conserved in other haptophyte algae including both alkenone-producing and non-alkenone-producing species (Fig. 4c). The presence of Akd1-homologous sequences in non-alkenone-producing genera such as *Pleurochrysis*, *Prymnesium* and *Chrysochromulina* suggests that the ancestral alkenone desaturase have existed before the genus differentiation in Haptophyta. How non-alkenone-producing species lost alkenone producing activity and obtained TAG-producing activity is very interesting in the evolution of haptophytes, but it remains to be elucidated in further study.

In this article, we reported a new transformation method optimised for an alkenone-producing haptophyte alga. Generally, heterologous expression system in bacteria or yeasts is the potent tool for gene functional analysis. In the case of alkenone metabolic pathway, however, applicability of this approach is quite limited, since non-alkenone-producing organisms cannot be used as a host. Therefore, we believe that this technique should provide opportunities for further studies on alkenone metabolism as well as other secondary metabolites.

We also presume that the novel alkenone desaturase Akd1 is a potential target for oceanic metagenomic and/or metatranscriptomic analyses, and will serve as a bridge between two currently independent research fields, contemporary algal biochemistry and archaeo-biogeochemistry. Further, *Akd1* and its homologous genes will be a useful tool to determine the ability of cold-tolerant feature and to elucidate cold-sensing molecular mechanisms in alkenone-producing haptophytes.

## Methods

### Algal Cells

The alkenone-producing haptophyte alga, *Tisochrysis lutea* (T-iso) (formerly *Isochrysis galbana* T-iso strain)^19,25^, kindly provided by Prof. T. Matsunaga of Tokyo University of Agriculture and Technology (Tokyo, Japan), was used in this study as describes in our previous report^34^. For this study, to select a high-alkenone-producing cell line, cells were separated on agar plate, and a single colony was isolated and established as a strain for further use.

### Growth conditions

Compositions of the culture medium and buffers are given in Supplementary Table 1. The artificial seawater-based medium (MA-ESM)^35^ were used for all the experimental cultures. The cells were grown in 500 mL flat-oblong glass vessels, with bubbling of filter-sterilised air at a rate of 100 mL/min. The culture was illuminated by continuous light from 18 or 36 W white fluorescent lamps at an intensity of 100 μmol photons m^−2^ s^−1^. The temperature was maintained at 20 °C, unless otherwise noted.

### Analysis of alkenones and alkenoates

Alkenone and alkenoates were analysed in the same way as described in our previous reports^23,36^. The analysis was performed on a Shimadzu GC-2014 GC-FID equipped with an Agilent CPSIL5CB column (50 m × 0.32 mm × 0.12 µm). The programs used for the separation of *T*. *lutea* and *E*. *huxleyi* alkenones were as follows: for *T*. *lutea*, 60 °C for 1.5 min, a gradient increase from 60 °C to 130 °C at 20 °C min^−1^ for 3.5 min, and a subsequent increase to 300 °C at 4 °C min^−1^ for 42.5 min followed by constant temperature at 300 °C for 25 min; for *E*. *huxleyi*, 80 °C for 3 min, a gradient increase from 80 °C to 180 °C at 15 °C min^−1^ for 6.6 min, a subsequent increase to 310 °C at 10 °C min^−1^ for 13 min followed by constant temperature at 310 °C for 20 min.

### Growth of *T*. *lutea* at different temperatures

Algal cells were grown in a 50 mL flask containing the MA-ESM at 15, 20, 25, and 30 °C for one week. The cells were then transferred to 500 mL glass vessels at the same temperatures and cultured for nine days under the same conditions. In all experiments, the cultures were continuously bubbled and illuminated by 18 or 36 W fluorescent lamps at an intensity of 100 μmol photons m^−2^ s^−1^. After sampling, TOC and alkenone contents were determined using a TOC-L analyser (Shimadzu Corporation) and GC-FID as describes in our previous report^34^. The data were obtained from four independent experiments.

### Expression analysis of *TOD-1* and *-2*

All primer sequences and PCR protocols are summarised in Supplementary Table 2. The complete ORF encoding TOD-1 and -2 was amplified with the following primer pairs; eORF2F1 and eORF2aR1 for *TOD-1*, and eORF2F1 and eORF2bR1 for *TOD-2* using KOD FX Neo DNA polymerase (Toyobo). Expression of *TOD-1* and -*2* was investigated by quantitative real-time PCR (qRT-PCR) as follows: (1) Total RNA was extracted from the cells using Trizol (Invitrogen) and the RNeasy Plant Mini Kit (Qiagen); the extracted RNA was treated with the TurboDNase Kit (Ambion). (2) Using 1 μg of total RNA as the template, cDNA was synthesised using the poly-T20 primer and SuperScriptIII reverse transcriptase (Invitrogen) in a 20 μL reaction mixture. (3) To examine the expression of *TOD-1* and -*2*, qRT-PCR was performed using 5 ng of cDNA as a template on the Applied Biosystems StepOnePlus Real-Time PCR System (ThermoFisher Scientific) using RT-qPCR SYBR GREEN Reagents (GoTaq qPCR Master Mix, Promega). As a reference, the expression of a house-keeping gene, *heat shock protein 70* (*Hsp70*), was also examined. Applied Biosystems StepOne Software v2.1 was used to operate the qRT-PCR system and to determine the crossing point for each amplification reaction. (4) The data were analysed using the ∆∆Ct method^37^ and normalised to *Hsp70* expression. For analysis of the overexpression mutant strains, the data were obtained from three independent experiments.

### Preparation of expression constructs

The promoter region of *Light-harvesting complex f17* (*Lhcf17*) was identified from a database containing a draft of the *T. lutea* genome (unpublished data). To construct the pLf17A7 vector that confers hygromycin B-resistance due to the presence of the *PyAph7* gene^29^, the endogenous *Lhcf17* promoter was inserted into the *Hin*dIII-*Xba*I site of the pFA7 vector^29^ (Supplementary Fig. 3a).

The tandem expression constructs (Fig. 3a), containing *TOD-1* and *PyAph7* were prepared as follows: (1) The *TOD-1* gene was subcloned into the *Xba*I-*Bam*HI site of the pLf17A7 vector. (2) The *Eco*RI-*Hin*dIII fragment containing the *PyAph7* cassette of pLf17A7 was extracted, blunt-ended, and subcloned into the *Nde*I site of the resultant vector. Constructs with the cassettes in the same or opposite orientations were designated as pLf17A7-TOD-1-F and -R, respectively.

### Gene transfer of *TOD-1* into *T. lutea* cells and isolation of transformed strains

The time and speed of centrifugation was fixed at 1,530 × *g* for 5 min. The cells in the late stationary phase (four to five-weeks after starting the culture at a density of 1.6–2.1 × 10^7^ cells/mL) were harvested from 25 mL of cell suspension by centrifugation. After washing cells with MA-ESM, the cells were resuspended in MA-ESM containing proteinase K (500 μg/mL), and incubated at 30 °C in a thermostatic water bath with moderate shaking for three to four hours. The cells were then collected by centrifugation, and resuspended in 12 ml of a hypo-osmotic buffer. The collected cells were transferred to a glass petri dish and incubated for 10 to 15 min. The cells were then filtered with a Miracloth (EMD Millipore, Darmstadt, Germany), centrifuged, and resuspended in 5 mL of a 0.4 M mannitol solution. After filtration through a tetron filter (180-mesh), approximately 3.2 × 10^6^ cells were resuspended in 320 μL of W5 buffer^29^. The removal of the cell wall was confirmed by calcofluor-staining (1% v/v).

Fifteen micrograms of the expression construct in 30 μL of Milli-Q water was added to the freshly prepared protoplast culture, and incubated in darkness for 10 min. The same volume (350 μL) of a 40% polyethyleneglycol (PEG) solution was added to the protoplast suspension and mixed, and incubated for 15 min. The 40% PEG solution was prepared by dissolving 2 g PEG (MW = 2,000, Wako) in 3 mL of CMS solution^29^. The PEG-treated cells were rinsed with 7 mL of MA-ESM and collected by centrifugation, then resuspended in MA-ESM. The cell suspension was incubated for three days and spotted onto a selective plate medium (0.2% gellan gum, 50% MA-ESM, and 1.0–1.5 mg/mL hygromycin B). The surviving colonies were picked and transferred to MA-ESM. Temperature and light intensity for growth were set at 25 °C and 60–100 μmol photons m^−2^ s^−1^ continuous illumination, respectively.

### Genotyping and expression analysis of the transformed strains

The insertion of *PyAph7* and *TOD-1* into the genome was confirmed by the analysis of genomic DNA extracted from the isolated transformed strains using the DNeasy Plant Mini Kit (Qiagen). The primers for the detection of *TOD-1* were designed to amplify the fragment containing the promoter region in order to avoid amplification of the endogenous gene (see Fig. 3a). As a control, *Hsp70* was also used as a target for amplification. PCR was performed using 1 or 10 ng of genomic DNA as the template, LA-Taq polymerase, and GC buffer 2 (TaKaRa Bio) in a 10 μL reaction.

For screening of *TOD-1*-harbouring transformed strains, the expression level of the gene was examined by reverse transcription PCR (RT-PCR). As an endogenous control, *Hsp70* was also amplified. Total RNA extraction and synthesis of cDNA was performed as described above (see “Expression analysis of *TOD-1* and *-2*”). The *TOD-1*, and *Hsp70* fragments were amplified by PCR using LA-Taq polymerase with GC buffer 2 using 50 ng of cDNA as a template in a 10 μL reaction. The amplified fragments were sequenced for both strands.

### Phylogenetic analysis of desaturases

The sequences of fatty acid desaturases of *E. huxleyi* and *P. tricornutum* were obtained from the databases of the Joint Genome Institute (https://genome.jgi.doe.gov/portal/). The gene IDs are listed in Supplementary Note. Desaturase sequences from a wide range of organisms were collected from the NCBI database (https://www.ncbi.nlm.nih.gov/). The deposited names (sequence IDs) are listed in Supplementary Note. All protein sequences were aligned using ClustalW^38^, and the phylogenetic tree was constructed using the maximum likelihood estimation program provided on IQ-Tree website with 1,000 bootstrapping steps^39^. Akd1-homologous sequences from *I. galbana* and *Gephyrocapsa oceanica* were identified by homology search from the transcriptome database on the website of iMicrobe (https://www.imicrobe.us/).

### Statistics

Analysis of variance (ANOVA) with a Tukey-test was used to compare the wild type and mutant strains, as shown in Fig. 3. In Fig. 3g, experimental groups with less than three samples were omitted from statistical analysis. A *t*-test was performed to compare the alkenone quantity before and after saponification, as shown in Supplementary Fig. 2b. In all cases, only two groups were analysed at the same time.

### Data availability

The sequences of promoter of *Lhcf17*, *TOD-1* and -*2* were deposited on the DDBJ database under the accession numbers, #LC336380, #LC336381, and #LC336382, respectively.

## Electronic supplementary material

Supplementary Information
