# Supplementary material for: Overexpression of Tisochrysis lutea Akd1 identifies a key cold-induced alkenone desaturase enzyme
Source: Sci Rep. 2018 Jul 25;8:11230. doi: 10.1038/s41598-018-29482-8 (PMC6060089; doi:10.1038/s41598-018-29482-8)
Supplement: Supplementary file 1 — Supplementary Information [file 41598_2018_29482_MOESM1_ESM.pdf]

Supplementary Information

Overexpression of *Tisochrysis lutea* Akd1 identifies a key cold-induced alkenone desaturase enzyme

Hirotoshi Endo, Yutaka Hanawa, Hiroya Araie[†], Iwane Suzuki & Yoshihiro Shiraiwa[*]

Faculty of Life and Environmental Sciences, University of Tsukuba, 1-1-1 Tennodai, Tsukuba, Ibaraki 305-8572, Japan.

[†]Present address: College of Science and Engineering, Kanto Gakuin University, 1-50-1 Mutsuura-higashi, Kanazawa-ku, Yokohama, Kanagawa 236-8501, Japan.

[*]Correspondence should be addressed to Y. S. (emilhux@biol.tsukuba.ac.jp)

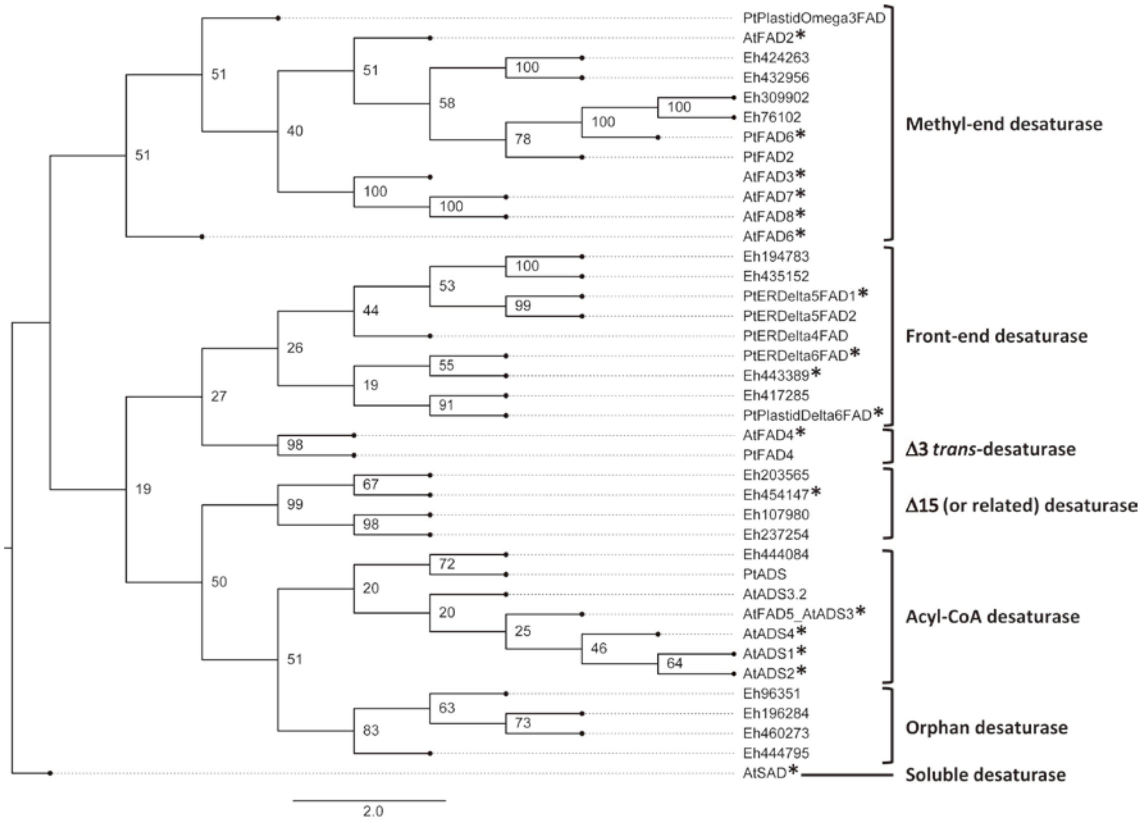

Supplementary Figure 1 | Identification of "orphan" desaturases in *Emiliania huxleyi*. The maximum likelihood phylogenetic tree of *cis*- and *trans*-fatty acid desaturases. Eh443389 and Eh454147 are the authentic Δ5 and Δ15 desaturases[17,22]. The four sequences in the orphan desaturase cluster were suggested to be candidates of alkenone desaturases. At, Pt and Eh: sequences from *Arabidopsis thaliana*, *Phaeodactylum tricornutum*, and *E. huxleyi*, respectively. *: functionally-characterised. Nomenclature of the sequences from *P. tricornutum*, according to Dolch et al.[26].

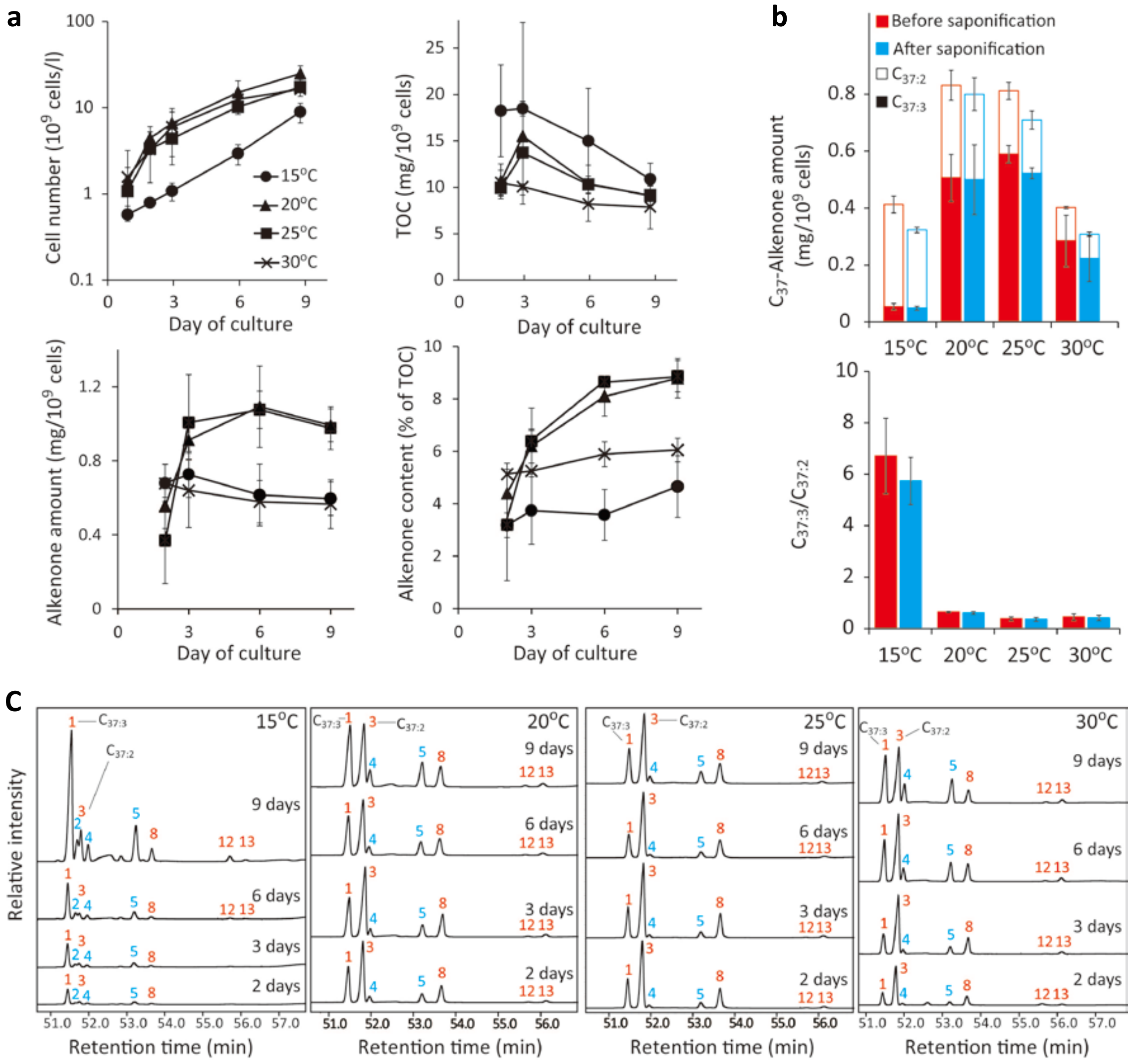

Supplementary Figure 2 | Cell growth, alkenone production, and unsaturation status in *Tisochrysis lutea* at different temperatures. a, Changes in cell number (*upper/left*), total organic carbon (TOC) content (*upper/right*), total alkenone content (*lower/left*), and alkenone content/TOC (*lower/right*). Data: mean ± s.d. (Day 2 at 30°C: n = 2, The others: n = 4). **b**, The $C_{37:3}$ and $C_{37:2}$ content (*upper*) and the $C_{37:3}/C_{37:2}$ ratio (*lower*) were analysed by GC-FID before and after saponification. Data: mean ± s.d. (n = 3). A *t*-test failed to detect any significant difference (p > 0.1). **c**, Partial GC-FID chromatograms of alkenones and alkenoates in samples without saponification. Peak numbers and colours are the same as shown in Fig. 1a.

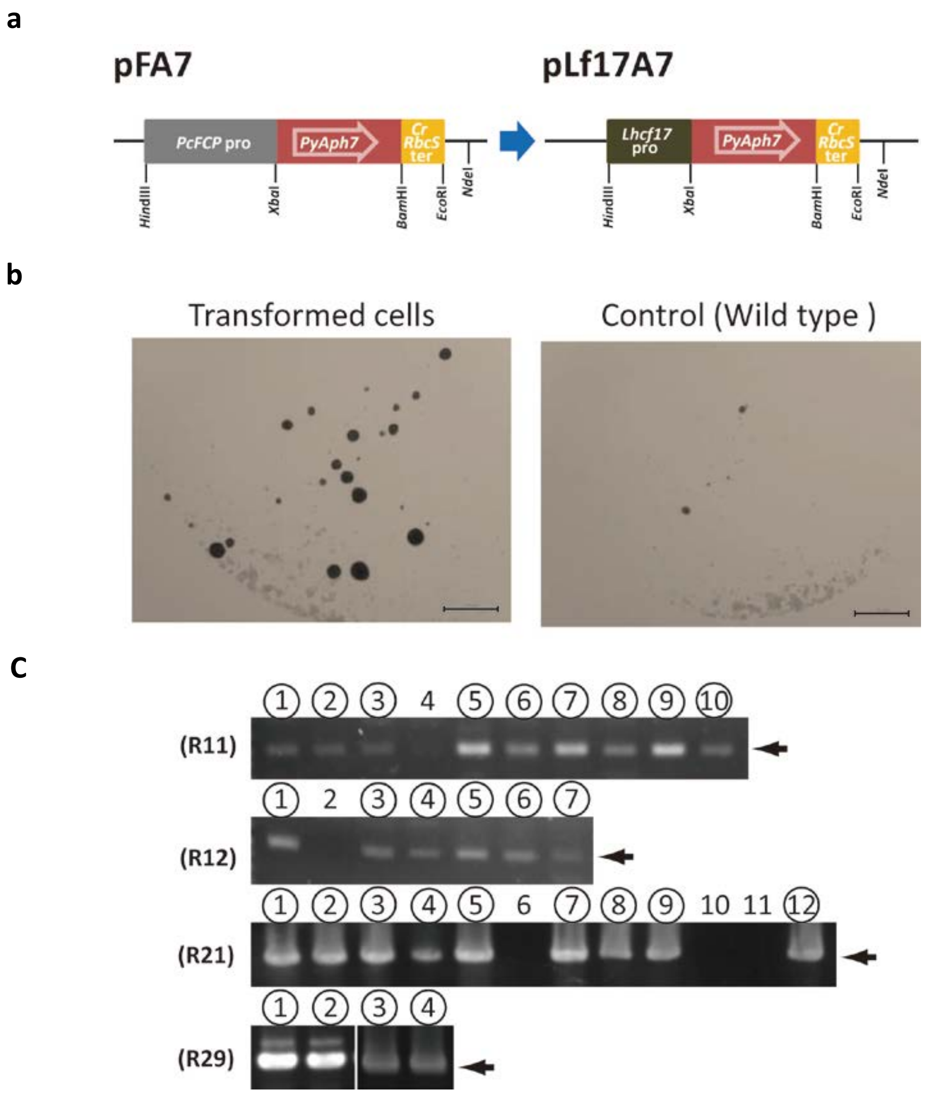

Supplementary Figure 3 | Generation of antibiotic-resistant mutant strains of *Tisochrysis lutea*. a, Hygromycin B-resistant construct used for *Pleurochrysis carterae* (pFA7, Endo et al.[29]) and the modified construct used for *T. lutea* (pLf17A7). **b**, Colonies of transformed (left) and wild type (right) cells on a hygromycin B selective plate. Bars: 1 mm. **c**, Genotyping of cells transformed with pLf17A7. The inserted *PyAph7* was detected by genomic PCR. The percentage of pseudo-positive colonies was approximately 12%. R-numbers in parentheses: Numbers of independent experiments. Numbers in circles above bands: Positive clones in the experiment. Arrows: Bands of the expected size. Original unmodified images of gel electrophoresis are shown in Supplementary Fig. 7.

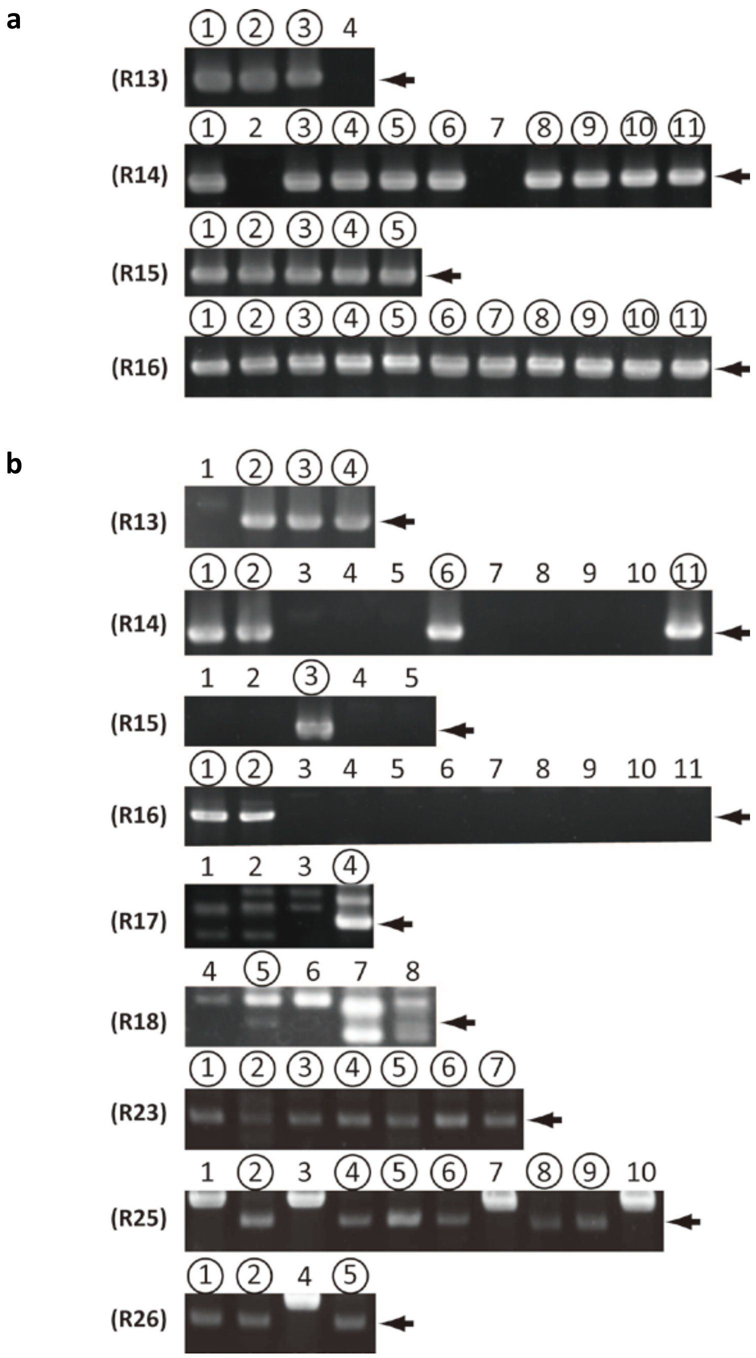

Supplementary Figure 4 | Genotyping of the cells transformed with the tandem expression constructs containing *PyAph7* and *TOD-1*. a and **b,** Genomic PCR detection of the inserted *PyAph7* and *TOD-1*, respectively. The primers for *TOD-1* were designed to amplify the fragment containing the promoter region in order to avoid amplification of the endogenous gene (see Fig. 3a and Methods). For R-numbers in parentheses, arrows and numbers above bands, see Supplementary Figure 3. Original unmodified images of gel electrophoresis are shown in Supplementary Fig. 7.

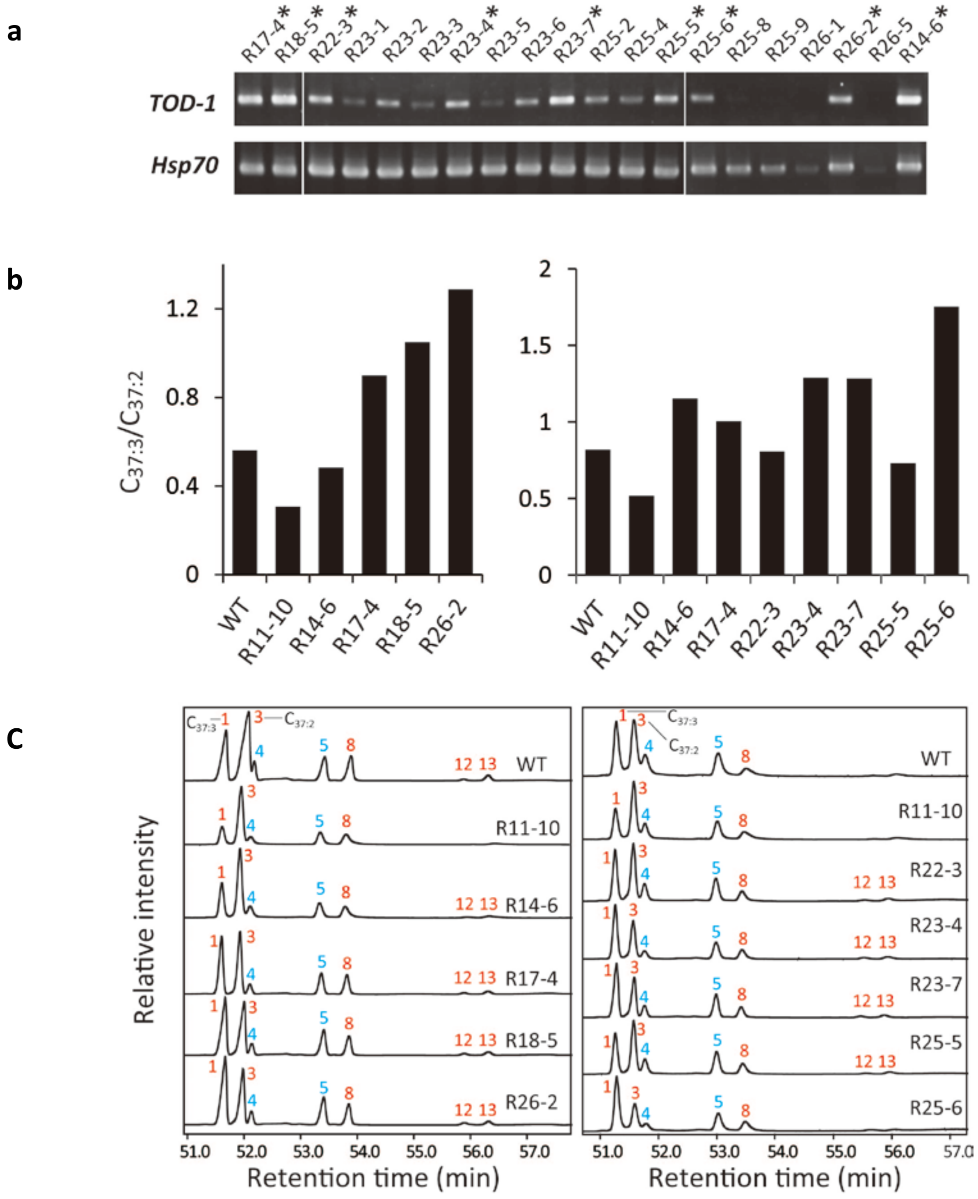

Supplementary Figure 5 | Screening of AtOM (<u>a</u>lkenone *<u>t</u>rans*-desaturase <u>o</u>verexpressing <u>m</u>utant) strains. a, Expression of *TOD-1* and a house-keeping gene *Hsp70* in the transformed strains. *: Strains showing relatively high expression of *TOD-1*. **b**, $C_{37:3}/C_{37:2}$ ratio from two independent experiments (*left* and *right*). **c**, Partial GC-FID chromatograms of alkenones and alkenoates extracted from wild type and the transformed strains from two independent experiments (*left* and *right*) (performed without saponification). Peak numbers and colours are the same as shown in Fig. 1a. WT and R-numbers: wild type strain and different transformed strains, respectively. Original unmodified images of gel electrophoresis are shown in Supplementary Fig. 7.

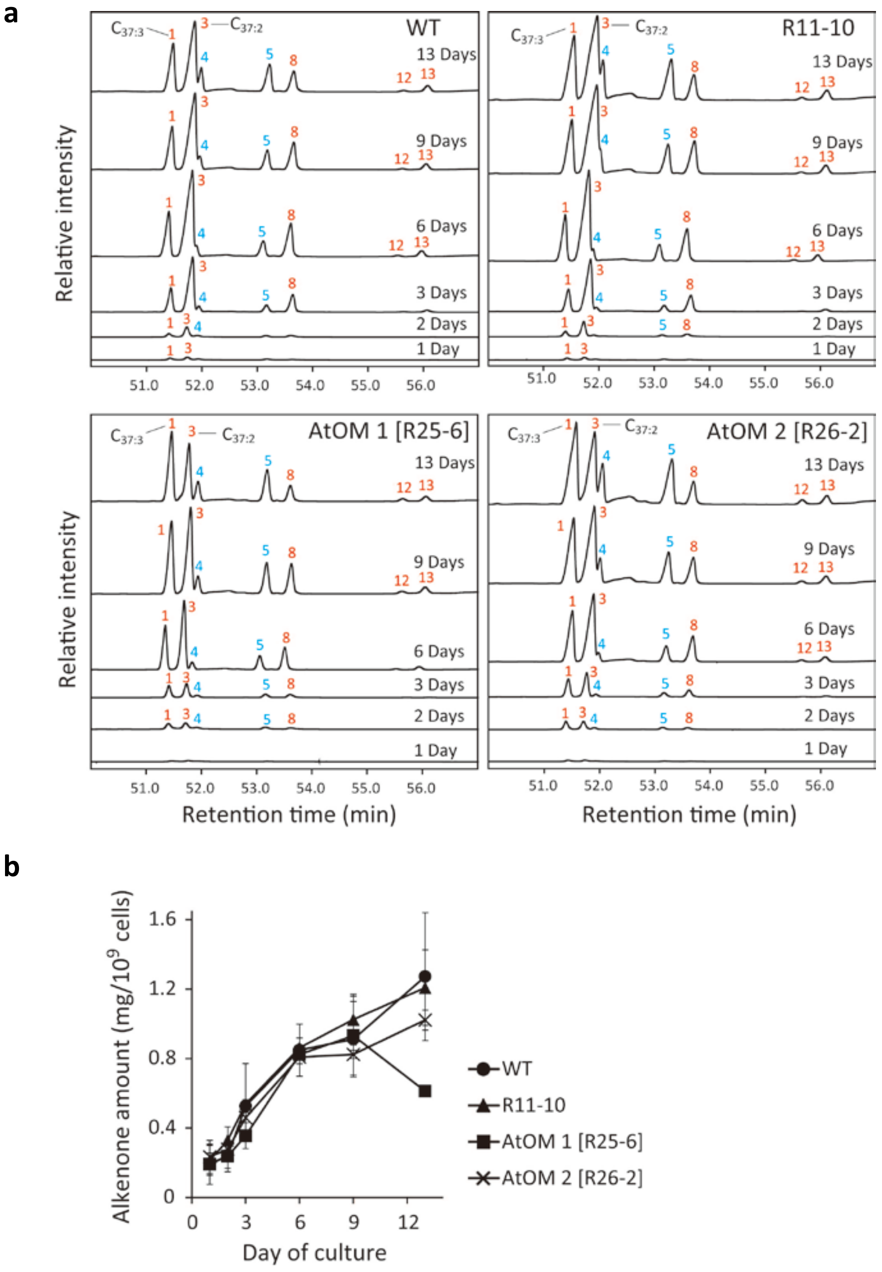

Supplementary Figure 6 | Alkenone production in the overexpressing mutant strains AtOM 1 and 2. a, Partial GC-FID chromatograms of alkenones and alkenoates were extracted from wild type and the transformed strains (performed without saponification). WT: wild type, R11-10: *PyAph7*-harboring control strain, AtOM 1 [R25-6] and 2 [R26-2]: alkenone *trans*-desaturase overexpressing mutant 1 and 2, respectively. The peak numbers and colours are the same as shown in Fig. 1a. **b**, Changes of total alkenone contents in the wild type and the transformed strains during culture for 13 days. Data: mean ± s.d.; n = 3.

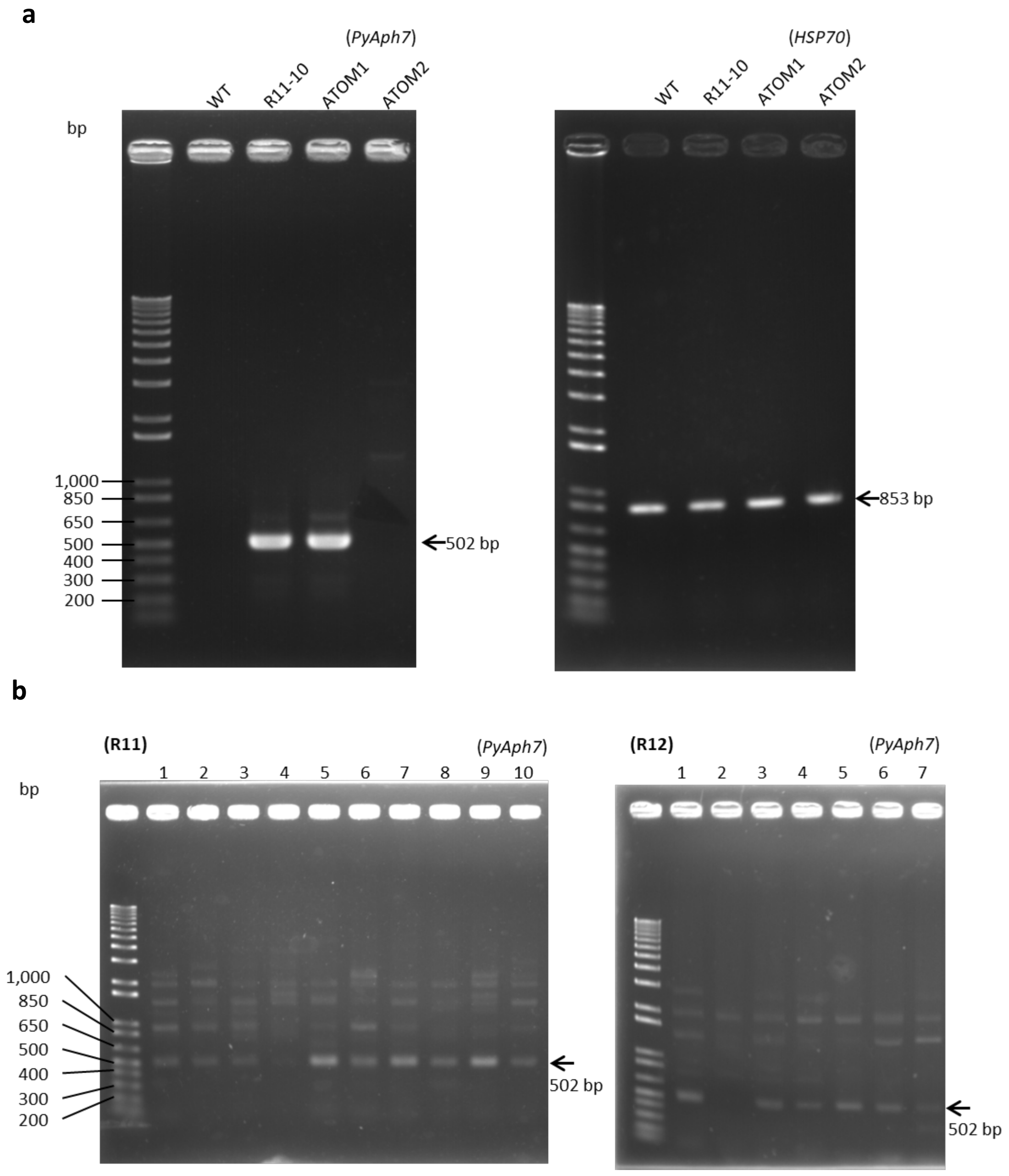
a
(PyAph7)
WT
R11-10
ATOM1
ATOM2
bp
1,000
850
650
500
400
300
200
502 bp
(HSP70)
WT
R11-10
ATOM1
ATOM2
853 bp
b
(R11)
(PyAph7)
1 2 3 4 5 6 7 8 9 10
bp
1,000
850
650
500
400
300
200
502 bp
(R12)
(PyAph7)
1 2 3 4 5 6 7
502 bp

b (continued)

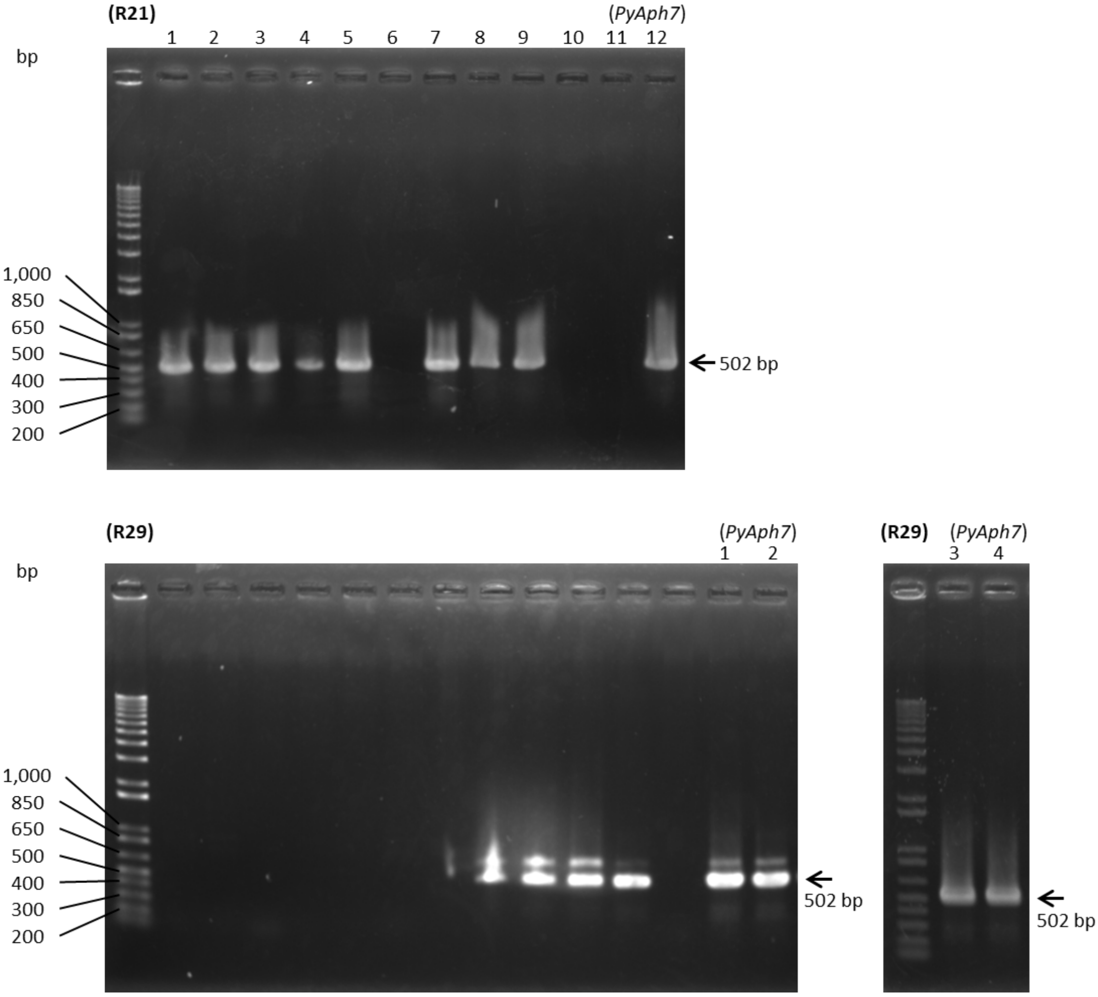
(R21)
1 2 3 4 5 6 7 8 9 10 11 12
(PyAph7)
bp
1,000
850
650
500
400
300
200
502 bp
(R29)
(PyAph7)
1 2
bp
1,000
850
650
500
400
300
200
502 bp
(R29)
(PyAph7)
3 4
502 bp

c

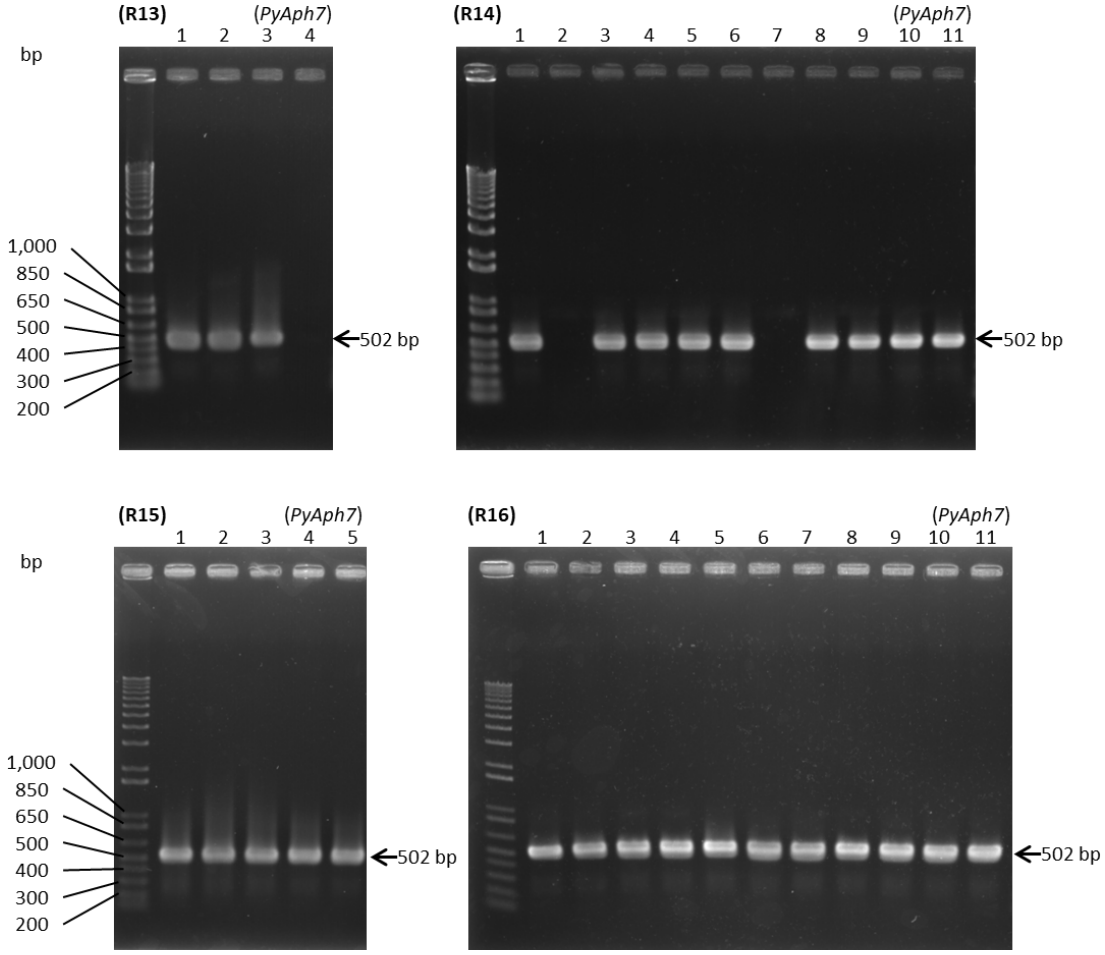
(R13)
(PyAph7)
1 2 3 4
bp
1,000
850
650
500
400
300
200
502 bp
(R14)
(PyAph7)
1 2 3 4 5 6 7 8 9 10 11
502 bp
(R15)
(PyAph7)
1 2 3 4 5
bp
1,000
850
650
500
400
300
200
502 bp
(R16)
(PyAph7)
1 2 3 4 5 6 7 8 9 10 11
502 bp

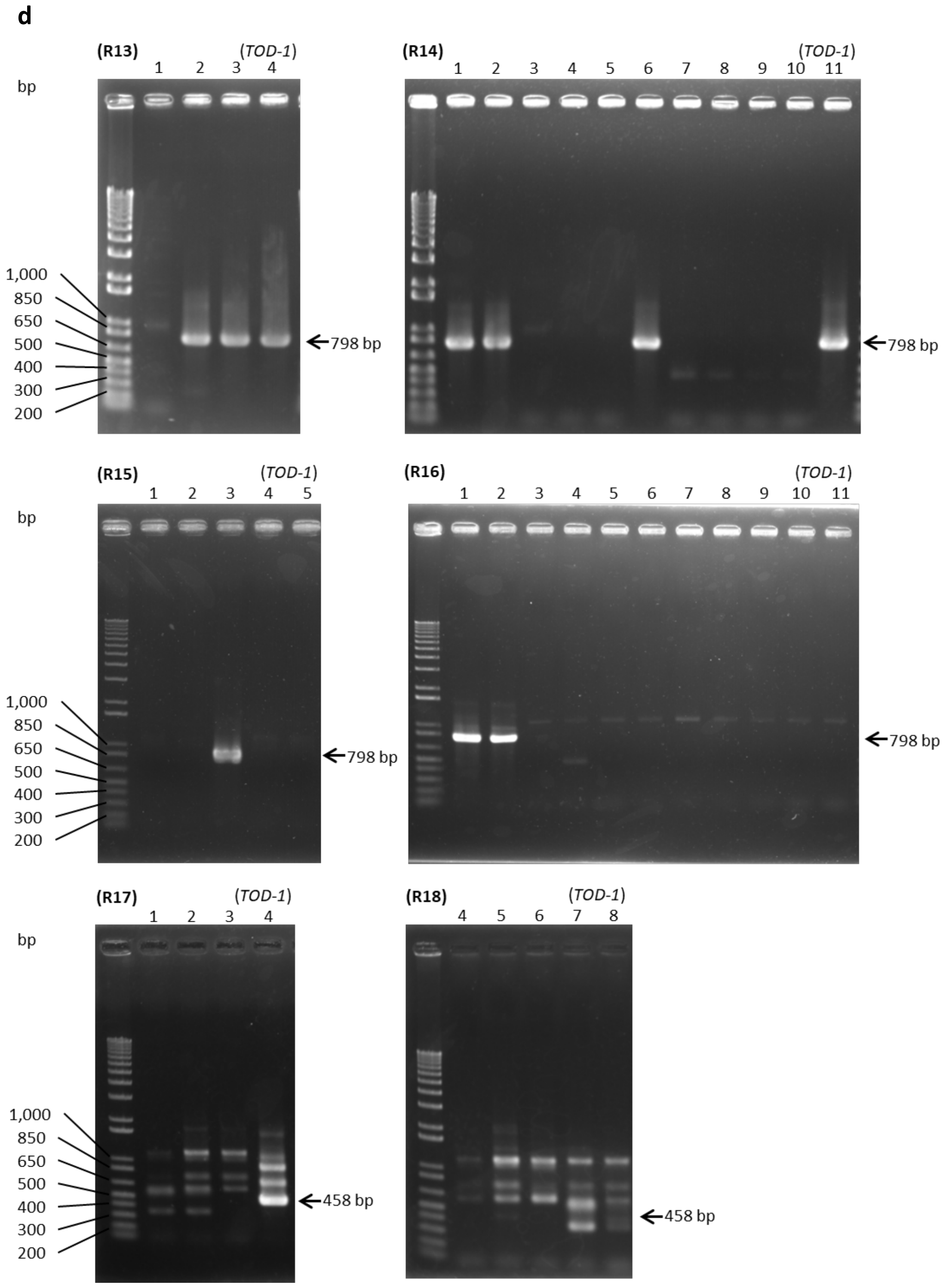
d
(R13)
(TOD-1)
1 2 3 4
bp
1,000
850
650
500
400
300
200
798 bp
(R14)
(TOD-1)
1 2 3 4 5 6 7 8 9 10 11
798 bp
(R15)
(TOD-1)
1 2 3 4 5
bp
1,000
850
650
500
400
300
200
798 bp
(R16)
(TOD-1)
1 2 3 4 5 6 7 8 9 10 11
798 bp
(R17)
(TOD-1)
1 2 3 4
bp
1,000
850
650
500
400
300
200
458 bp
(R18)
(TOD-1)
4 5 6 7 8
458 bp

d (continued)

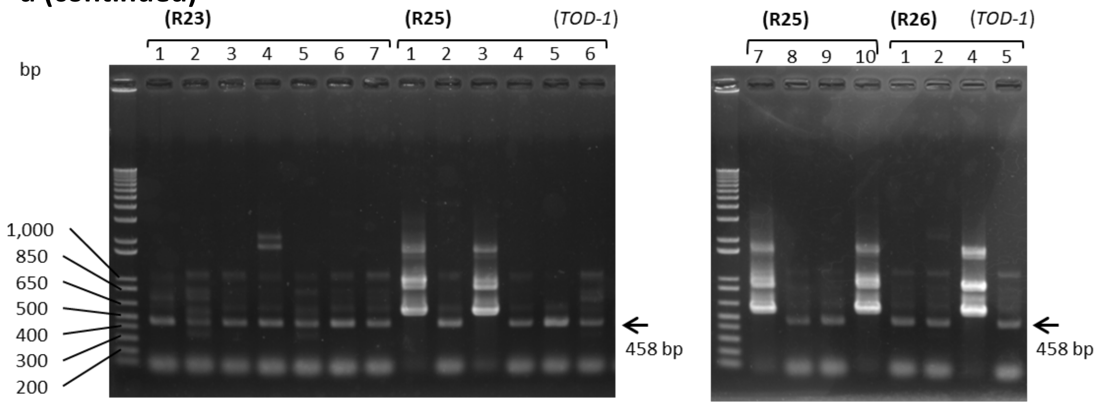

e

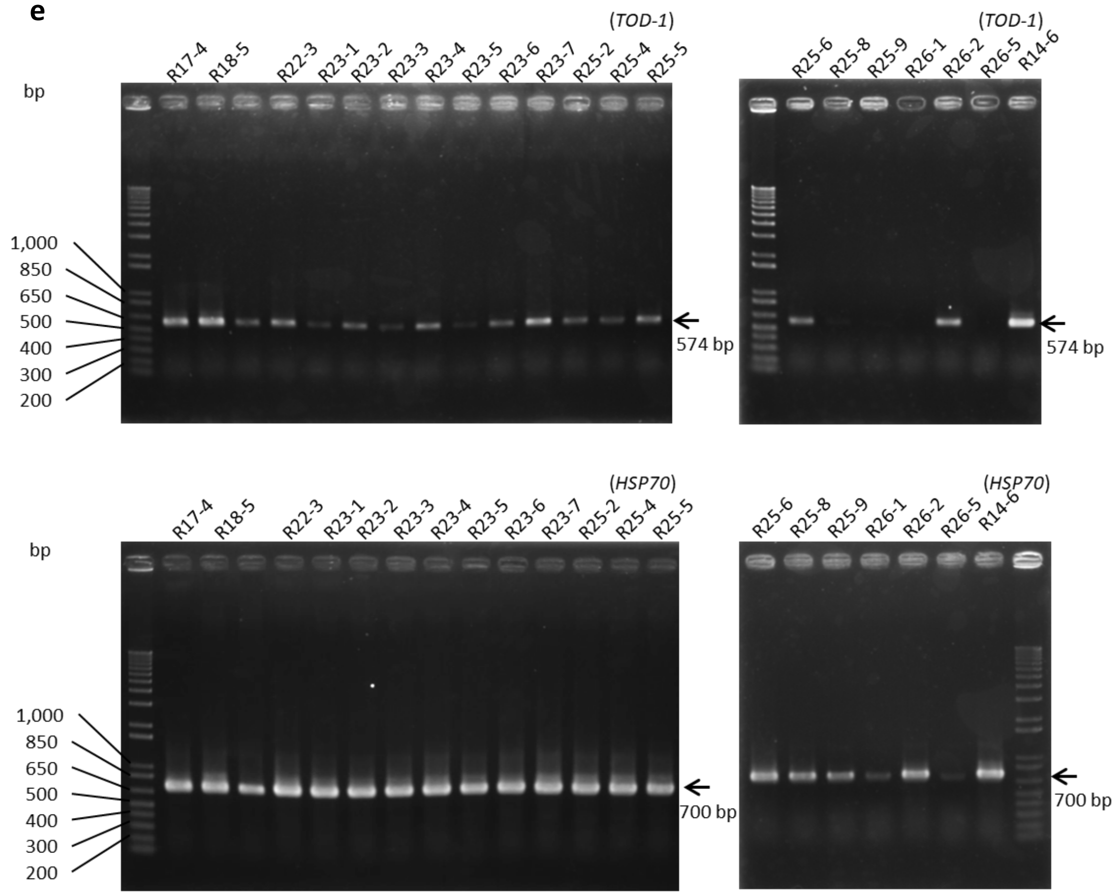

Supplementary Figure 7 | Original unmodified digital images of gel electrophoreses. a, Original images of Fig. 3b. **b**, Original gel images of Supplementary Fig. 3c. **c** and **d**, Original gel images of Supplementary Fig. 4a and 4b, respectively. **e**, Original gel images of Supplementary Fig. 5a.

Supplementary Table 1 | Culture media and solutions used in the present study.

(a) MA-ESM*			
Marine Art SF1		modified ESM	
Salinity	30‰	$NaNO_3$	0.12 g/L
NaCl	22.1 g/L	K_2HPO_4	5 mg/L
$MgCl_2 \cdot 6H_20$	9.9 g/L	Tris	1 g/L
KCl	0.61 g/L	Fe-EDTA	259 µg/L
$CaCl_2 \cdot 6H_20$	1.5 g/L	Mn-EDTA	332 µg/L
$NaHCO_3$	0.19 g/L	Thiamin·HCl	100 µg/L
KBr	96 mg/L	Biotin	1 µg/L
Na_2SO_4	3.9 g/L	Cyanocobalamin	1 µg/L
LiCl	1 mg/L	Na_2SeO_3	1.7 µg/L
$AlCl_3 \cdot 6H_20$	8 µg/L		
$FeCl_3 \cdot 6H_20$	5 µg/L		
$MnCl_2 \cdot 4H_20$	0.6 µg/L		
$CoCl_2 \cdot 6H_20$	2 µg/L		
$SrCl_2$	13 mg/L		
$Na_2B_4O_7 \cdot 10H_20$	78 mg/L		
NaF	3 mg/L		
KI	81 µg/L		
$Na_2WO_4 \cdot 2H_20$	2 µg/L		
$(NH_4)_6Mo_7O_{24} \cdot 4H_20$	18 µg/L		

(b) hypo-osmotic buffer	
HEPES	10 mM
KCl	100 mM
NaOH	350 µM

(c) W5 buffer	
MES	2 mM
NaCl	154 mM
$CaCl_2$	125 mM
KCl	5 mM
pH	5.7

(d) CMS solution	
Mannnitol	400 mM
$Ca(NO_3)_2$	100 mM
pH	7.0

*Tomita Pharmaceutical Co. Ltd.

Supplementary Table 2 | Primers and PCR conditions used in the present study.

Sequence of primers				
Gene	Primer name	Primer (5'→3')	Reaction	Aim
PyAph7	PyAph7_F-T_Iso	GACGCAGGAGTCCCTGCT	1st PCR	Genome insertion check (Genomic PCR) Expression analysis (RT-PCR)
	PyAph7_R-T_Iso	ACGAAGATGTTGGTCCCGT		
	PyAph7_FN-T_Iso	GCTCGACCGCATTGACTC	Nested PCR	
	PyAph7_RN-T_Iso	CAGCTCCGGGAAGACCTC		
Hsp70	HSP70_F-T_Iso	GGTCGACACAGTCATTGGAA	Genomic PCR	Genome insertion check
	HSP70_R-T_Iso	GCGATTGTCGAAGTCCTCTC	RT-PCR	Expression analysis
	IsoRT-HSP70-F	GCTCCACTCGCATTCCCAAG	RT-qPCR	Expression analysis
	IsoRT-HSP70-R	GTCTCCTCGCCACCCTCAC		
TOD-1	eORF2F1	GTCGCGGCCGCCCTCCCACT	RT-PCR$^{(1)}$	Cloning and sequence
	eORF2aR1	AGTGAGCGGTACCTTGATGG		
	Lhcf17-UNI_F1	CCAAAAGGGGCTCTCCTAGT	1st PCR	Genome insertion check (Genomic PCR)
	Lhcf17-DES1_R1	AATCAGAGGGAACACGAACG		
	Lhcf17-UNI_F2	TCACTTCATCTGTCCGCAAA	Nested PCR	
	Lhcf17-DES1_R2	AAGGTGGTGTCGCAAAACTT		
	Lhcf17-DES1-F	TCTGCATGAGCGTAATCCTG	RT-PCR$^{(2)}$	Expression analysis
	Lhcf17-DES1-R	GAATCGATGGTCGTTGGTTT		
	Des1_RT-4NF	AAGCTTTAGCTCGTCATGGATC	RT-qPCR	Expression analysis
	Des1_RT-4NR	GGCGATGAATCGATGGTC		
TOD-2	eORF2F1	GTCGCGGCCGCCCTCCCACT	RT-PCR	Cloning and sequence
	eORF2bR1	ATGGCGCTCGTCGTACAT		
	Des1_H_RT-NF	CATGGCCTCACCTCGAAATA	RT-qPCR	Expression analysis
	Des1_H_RT-NR	GTCGCCACAAGAAATGCCAG		
PCR programs				
PyAph7	Genomic PCR/RT-PCR			
	1st PCR	94°C 2 min, (94°C 30 s, 58°C 30 s, 72°C, 45 s) × 35, 72°C 5 min		
	Nested PCR	94°C 2 min, (94°C 30 s, 58°C 30 s, 72°C, 30 s) × 30, 72°C 5 min		
	RT-qPCR	95°C 2 min, (95°C 15 s, 60°C 60 s) × 40, 95°C 15 s, 60°C 1 min, 95°C 15 s		
Hsp70	RT-PCR	94°C 2 min, (94°C 30 s, 58°C 30 s, 72°C, 45 s) × 27, 72°C 5 min		
	Genomic PCR	94°C 2 min, (94°C 30 s, 58°C 30 s, 72°C, 60 s) × 27, 72°C 5 min		
	RT-qPCR	95°C 2 min, (95°C 15 s, 60°C 60 s) × 40, 95°C 15 s, 60°C 1 min, 95°C 15 s		
TOD-1	Genomic PCR			
	1st PCR	94°C 2 min, (94°C 30 s, 58°C 30 s, 72°C, 75 s) × 35, 72°C 5 min		
	Nested PCR	94°C 2 min, (94°C 30 s, 58°C 30 s, 72°C, 30 s) × 30, 72°C 5 min		
	RT-PCR$^{(1)}$	94°C 2 min, (98°C 10 s, 68°C 50 s) × 35		
	RT-PCR$^{(2)}$	94°C 2 min, (94°C 30 s, 58°C 30 s, 72°C, 45 s) × 30, 72°C 5 min		
	RT-qPCR	95°C 2 min, (95°C 15 s, 60°C 60 s) × 40, 95°C 15 s, 60°C 1 min, 95°C 15 s		
TOD-2	RT-PCR	94°C 2 min, (98°C 10 s, 68°C 50 s) × 35		
	RT-qPCR	95°C 2 min, (95°C 15 s, 60°C 60 s) × 40, 95°C 15 s, 60°C 1 min, 95°C 15 s		

Supplementary Note | Sequence IDs used in phylogenetic analyses.

All the sequences from *E. huxleyi* or *P. tricornutum* were deposited under '*Emihu1*' or '*Phatdraft*' accessions, respectively. For *P. tricornutum*, the gene IDs of the sequences were PtADS (Phatdr_28797), PtERDelta5FAD1 (Phatdr_46830), PtERDelta5FAD2 (Phatdr_22459), PtERDelta6FAD (Phatdr_2948), PtFAD2 (Phatdr_25769), PtFAD4 (Phatdr_41301), PtFAD6 (Phatdr_48423), PtPlastidDelta6FAD (Phatdr_50443), PtERDelat4FAD (Phatdr_22510), and PtPlastidOmega3FAD (Phatdr_41570). For genes from *P. tricornutum*, the nomenclature was according to Dolch et al[26]. The FAD sequences of *Arabidopsis thaliana* were retrieved from the NCBI database. The Sequence IDs deposited were AtADS1 (NP_172098), AtADS2 (NP_565721), AtADS3.2 (NP_188208), AtADS4 (NP_172124), AtFAD2 (NP_187819), AtFAD3 (NP_180559), AtFAD4 (NP_194433), AtFAD5/AtADS3 (NP_566529), AtFAD6 (NP_194824), AtFAD7 (NP_187727), AtFAD8 (NP_196177), and AtSAD (NP_181899).

Desaturase sequences from a wide range of organisms were collected from the NCBI database. The deposited names (sequence IDs) were as follows; for Δ9 desaturases: *Amylomyces_rouxii*_d9 (AAB82294), *Antheraea_pernyi*_d9 (ADO85598), *Arabidopsis_thaliana*_d9 (AAM63359), *Mortierella_alpina*_d9 (CAB38177), *Rattus_norvegicus*_d9 (Q6P7B9), *Saccharomyces_cerevisiae*_d9 (NP_011460), *Xenopus_laevis*_d9 (NP_001087809), and *Yarrowia_lipolytica*_d9 (XP_501496); for Δ11 trans-desaturases: *Epiphyas_postvittana*_d11 (AAL11496); for Δ12 desaturases: *Amylomyces_rouxii*_d12 (AAD55982), *Aspergillus_flavus*_d12 (XP_001818769), *Borago_officinalis*_d12 (AAC31698), *Caenorhabditis_elegans*_d12 (NP_502560), *Chlorella_vulgaris*_d12 (BAB78716), *Helianthus_annuus*_d12 (XP_022000024), and

*Trypanosoma_equiperdum*_d12 (XP_951577); for Δ4 desaturases: *Isochrysis_galbana*_d4 (AFD22891), *Pavlova_viridis*_d4 (ADG36330), *Thalassiosira_pseudonana*_d4 (AAX14506), and *Thraustochytrium*_sp_d4 (AAZ43257); for Δ5 desaturases: *Caenorhabditis_elegans*_d5 (NP_001255423), *Homo_sapiens*_d5 (AAF29378), *Mortierella_alpine*_d5 (BAD95486), *Octopus_vulgaris*_d5 (AEK20864), *Pavlova_salina*_d5 (A4KDP0), *Pythium_irregulare*_d5 (AAL13311), and *Rattus_norvegicus*_d5 (NP_445897); for Δ6 desaturases: *Borago_officinalis*_d6 (AAD01410), *Homo_sapiens*_d6 (NP_004256), *Mortierella_alpine*_d6 (CAE53093), *Mucor_circinelloides*_d6 (BAB69055), *Pythium_irregulare*_d6 (AAL13310), *Rachycentron_canadum*_d6 (ACJ65149), *Rattus_norvegicus*_d6 (NP_112634), and *Rhizopus_stolonifera*_d6 (ABB96724); for Δ3 *trans*-desaturases: *Arabidopsis_thaliana*_d3 (NP_194433); for Δ4 sphingolipid desaturases: *Drosophila_melanogaster*_d4Sphingo (NP_476594), *Homo_sapiens*_d4Sphingo (NP_003667), and *Mus_musculus*_d4Sphingo (AAM12532); for Δ8 sphingolipid desaturases: *Arabidopsis_thaliana*_d8Shingo (NP_191717) and *Brassica_napus*_d8Sphingo (NP_001302507).
